# Relocation experiences of the elderly to a long-term care facility in Taiwan: a qualitative study

**DOI:** 10.1186/s12877-020-01679-5

**Published:** 2020-08-06

**Authors:** Chia-Shan Wu, Jiin-Ru Rong

**Affiliations:** 1 Department of Nursing, National Tainan Junior College of Nursing, 78, Sec.2 Minzu Rd, Tainan City, Taiwan; 2 School of Nursing, National Taipei University of Nursing and Health Sciences, 365, Ming-Te Road, Peitou, Taipei, Taiwan

**Keywords:** Elderly well-being, Relocation stress, Long-term care facility

## Abstract

**Background:**

Relocation to a long-term care (LTC) facility is a major life change for most elderly people. Following relocation, many elderly experience difficulties in adapting to changes in the living environment. Taiwan is increasingly becoming an “aging society” and the numbers of those who relocate from family residences to long-term residential care facilities have increased over years. However, in-depth evidence on the experiences of the elderly of their stay in LTC facilities in Taiwan is relatively sparse. This study aimed to explore the relocation experiences of the elderly to a LTC facility to inform policy and practice to address their needs effectively.

**Methods:**

A qualitative study, using semi-structured in-depth interviews, was conducted to explore the experiences of 16 elderly people who have relocated to and lived in a LTC facility in Taiwan for up to a period of 12 months. All interviews were recorded, transcribed, and analyzed using grounded theory approach.

**Results:**

Participants’ accounts reflected four interrelated key themes: wish to minimize the burden, but stay connected with the family; perceived barriers to adaptation; valuing tailored care; and acceptance and engagement. Each theme included interrelated subthemes that influenced one another and represented the different stages in the relocation journey. Most participants viewed relocation as a way of minimizing the burden of their care from family members, but desired to keep a close connection with family and friends. Participants recounted experiences of psychological resistance while making the decision to relocate. Fear of losing autonomy and the ability to perform self-care was a major reason for resistance to adapt. Provision of tailored care was accorded much value by the participants. The decision to accept the relocation and to adapt themselves to the new environment due to their needs for constant care was explicit in some accounts.

**Conclusions:**

Relocation to LTC facility is a dynamic process in the first year of moving into the facility, and involves a range of emotions, feelings and experiences. Adaptation of the elderly into the LTC facility can be maximized if the relocation is well planned with provisions for individually tailored care and family involvement.

## Background

With an ever growing ageing population, the care of the elderly is at the forefront of policy and practice discussions worldwide. Globally approximately 901 million people were estimated to be aged 60 years and over in 2015, representing 12.3% of the population [1]. The current pace of population ageing is postulated to be faster than in the past and the proportion of those aged over 60 years worldwide is likely to double from 12 to 22% between 2015 and 2050 [2]. There is a recognition of the impact of the aging population in many countries as evidenced by increases in both community and residential home based care facilities for the elderly.

Residential care facilities providing housing, care and other supportive and rehabilitative services tend to be a sought after option for the elderly who are unable to live independently. Long-term relocation to a residential care facility could occur due to a number of reasons, including the levels of dependency on others for routine activities, lack of adequate cognitive capacity, need for specialized care that cannot be provided in community-based settings, lack of social support networks and/or the inability of family members or others to care for the individual in a non-institutional setting [3]. While long-term residential care often provides a range of personal or health care services, evidence on the impact of relocation to and between residential care facilities appears to be inconclusive with researchers reporting both positive and negative outcomes overall [4–7].

Relocation to a long-term care facility (LTC) tend to be a major life change for most elderly with ensuing changes in relationships with their family and friends. Studies, mainly conducted in USA, Europe and Australia, have examined the settling in or adjustment of the elderly into care facilities and existing reviews have synthesized their key findings [8–12]. For example, in their review of the views and experiences of the elderly following relocation to residential care, Lee et al. (2002) reported coping strategies used by the elderly including passive acceptance, making the best of available choices, and reframing [8, 9]. While qualitative reviews are relatively sparse, Sullivan and Williams (2017) have meta-synthesised the findings of studies conducted in USA or Canada on the transition experiences of the elderly who have recently relocated to LTC facilities and reported three overall themes: painful loss requiring a mourning process, seeking stability through gaining autonomy to sustain a new sense of self, and acceptance when a unique inner balance is reached [11]. A recent mixed method narrative synthesis mapped potential personal and community focused facilitators and inhibitors in elderly people’s transition to LTC facilities to four themes including individual resilience, interpersonal connections and relationships, the feelings around the LTC facility as the new home, and the care facility as an organisation [12].

Many elderly people experience difficulties in adapting to changes to their physical living environment as well as changes to their activities of daily living, and social networks following relocation [13, 14]. Researchers have come up with different terminologies such as relocation stress syndrome, transfer trauma, and relocation syndrome to indicate the negative psychological impact of relocation [15–17]. Reported adverse consequences of relocation include decline in functional and cognitive capacity and general wellbeing, increased feelings of loneliness, accidental falls and injuries, and increased mortality and morbidity [18–22]. It been indicated that most of the psychological impact of relocation tend to manifest within the first 6 months of stay in the facility and the impact varies with the physiological and psychological state of the person [23–25]. The emotional distress increases with age, and tend to manifest more in elderly people aged more than 80 years [26]. Although less frequent, studies have reported positive outcomes such as enhanced engagement and participation in social activities and improvements in emotional wellbeing [26–28].

With about 14.85% of the population aged 65 years or older, Taiwan is increasingly seen to be an “aging society” [29]. In traditional Asian Chinese communities, filial piety, the children’s duty of care for parents and older family members, is an important norm that governs the care of the elderly parents and relatives and adult children are normally expected to care for and serve their parents in old age [30]. Similar norms are upheld in Taiwan as a traditional Chinese society. While most of the elderly people in Taiwan live in the community supported by their families, the numbers of those who have relocated from family residences to long-term residential care facilities have increased over the past decade due to factors such as urbanization, changes in family structure, longer life spans, and changing patterns of illness among the elderly [18, 31–33]. LTC facilities in Taiwan include assisted living facilities and nursing homes that provide the rehabilitative, restorative, and/or ongoing skilled nursing care needed in general or in relation to specific health conditions. The nursing homes offer health care services, medical care and skilled nursing care for residents who have seriously ill and/or need long-term care for chronic diseases. Some nursing homes also provide services such as physical therapy, occupational therapy, or speech-language therapy. An assisted living facility provides care for people who cannot or choose not to live independently, care services offered include: assistance with daily living activities (bathing, dressing, eating, toileting, etc.), dining programs that include three meals a day, and group activities. However, relocation to an LTC facility can be viewed by both the elderly and their families in Taiwan as a contradiction to the cultural expectation and a violation of children’s filial piety obligations in Taiwan. In a qualitative study published a decade ago, Wu et al. (2009) found that the care of the elderly in LTC facilities is seen to be a process of forced choice involving three stages: ‘becoming a problem’, ‘making a forced choice’ and ‘coping with the forced choice’ [34]. Another study that explored the culture of LTC facilities for the elderly in Taiwan reported themes including ‘collective life’, ‘care rituals’ and ‘embedded beliefs’ concluding that the elderly experienced a tedious, monotonous, idle and lonely life in the facilities [35]. Other studies have indicated factors that influence the expectations of long-term residential care among Taiwanese elderly [18, 31–33]. For example, a study that investigated the preferences of the elderly in northern Taiwan with regard to various types of long-term care services found that ethnic background and the requirement for additional medical care services had significant impact on the long-term care preferences [32]. However, recent in-depth qualitative evidence on the experiences of the elderly of their stay in LTC facilities is relatively sparse in Taiwan. The aim of this study was to explore the relocation experiences of the elderly to LTC facilities in Taiwan to inform policy and practice to address the needs effectively. The focus of the study was on the experiences of the elderly who have lived in the facility for up to a period of 12 months.

## Methods

A qualitative study based on a grounded theory approach [36] was conducted to explore the experiences of the elderly during the first year of their stay in LTC facilities in Taiwan.

### Recruitment of participants

The participants were 16 elderly people who have relocated to two LTC facilities in Taiwan and lived in the facility for up to 12 months. The criteria for selection of the LTC facilities was that they provide nursing as well as residential care facilities and were accessible to the researchers. The inclusion criteria for participants were that they: (1) were aged 65 years or over, (2) relocated and lived in a LTC facility for up to 12 months, (3) were conscious and alert, and can communicate continuously for 30 min, (4) agreed to participate in the interview, (5) did not have a history of alcoholism, drug addiction, dementia, severe cognitive impairment or other diagnosed mental illnesses and (6) did not have severe hearing or communication problems.

After gaining relevant ethics approval from the Ethics Committee of National Cheng Kung University (NCKU HREC-E-106-230-2), the researchers contacted nurses at two LTC facilities in Tainan, Taiwan. The researchers made initial contact with the participants after potential participants were personally introduced by the nurses in charge in the facilities. During the first visit, the researcher described the purpose and what the participation involved. Those who expressed an interest to participate after the initial discussion were given detailed information about the study. The researchers obtained written consent before enrolling the participants in the study.

### Data collection

Consistent with the objectives of the study, a semi-structured topic guide was developed based on an extensive review of the literature along with discussions with experts in the field. The literature review was instrumental in shaping the overall research question, identifying the key issues reported internationally and to tap them into culturally sensitive questions to understand the experiences of the participants. Broadly, the topic guide included questions on the reasons participants decided to move to a LTC facility, their experiences of relocation to the facility, their day-to-day activities, their likes and dislikes about the facility, and their reasons for continuing to live in the facility (Additional file 1). The face to face in-depth interviews took place in a quiet private room in the facility at a mutually convenient prearranged time. There was no one else present apart from the researcher and the participant during the interview, and the participants were encouraged to speak freely. All of the interviews were conducted by the first author (C-S Wu).

The interviews lasted between 60 and 90 min. To ensure credibility and to facilitate participants sharing their real-life experiences, the interviews were conducted either in Mandarin and Taiwanese as desired by participants. The researchers assured the participants that anything they said would be valued and kept in strict confidence. The researcher audio-recorded the interviews with permission from participants and took notes during the interviews. Data collection in this study continued until data saturation was reached when no new themes or concepts related to the topic emerged.

### Analytical approach

A professional transcriptionist transcribed verbatim all of the audio recordings of interviews soon after the interview. The researcher checked the contents of the transcript within a day to prevent researcher memory bias. A continuous comparative analysis was adopted for analysis with the researchers starting the data analysis soon after the first interview and the subsequent analysis being performed simultaneously with the data collection [36]. The analysis was done manually. The first stage of the analysis involved close reading of the interview transcripts several times to familiarize and identify the key themes emerging from the interviews. A line-by-line coding of the transcripts using a three-stage coding process involving open coding, axial coding and selective coding was performed [36, 37] to identify and name concepts and categories and to determine their relationships. The coding and categorization was done using the computer program Word. The analysis was conducted by the two researchers with background in Nursing and experience in qualitative analysis. To minimize bias and maintain objectivity, the authors regularly. Discussed and evaluated the interview procedures, as well as compared, and jointly conceptualized the findings. The study’s scientific rigor was enhanced throughout the research process by constant evaluation of credibility, dependability, transferability, and confirmability [38, 39]. The interviewers remained neutral and objective throughout the interview process by actively avoiding subjective judgments and encouraging participants to describe their experiences in detail.

## Results

The characteristics of the 16 participants are presented in Table 1. The average age was 81.9 years with the majority (11 participants) aged over 80 years. All the participants had at least two illnesses or chronic conditions. The participants generally had the ability to take care of themselves, but were unable to do activities such as cooking, cleaning the room or washing their clothes or take medication regularly. All the participants lived in shared rooms with other residents. The average length of stay in the LTC facility was 5.6 months with the majority (10 participants) living in the facility for less than 6 months.

**Table 1:** Participant characteristics

Participants	R1	R2	R3	R4	R5	R6	R7	R8	R9	R10	R11	R12	R13	R14	R15	R16
Age group	81–85	86–90	86–90	65–70	76–80	76–80	86–90	86–90	86–90	76–80	86–90	81–85	81–85	65–70	71–75	86–90
Gender	Male	Male	Female	Male	Female	Female	Female	Female	Female	Female	Male	Female	Female	Female	Female	Male
Diagnosis	Cancer	Hypertension	Hypertension	Hypertension	Hypertension	Cancer	Hypertensive Heart Disease	Diabetes mellitus	Parkinson disease	Hypertension	Hypertension	Hypertensive Heart Disease	Cardiovascular Accident	Diabetes mellitus	Parkinson disease	Diabetes mellitus
Living situation	Nursing Home	Nursing Home	Assisted-Living facility	Assisted-Living facility	Assisted-Living facility	Assisted-Living facility	Nursing Home	Nursing Home	Assisted-Living facility	Assisted-Living facility	Assisted-Living facility	Nursing Home	Nursing Home	Assisted-Living facility	Assisted-Living facility	Assisted-Living facility
Length of stay	5 Months	8 Months	9 Months	10 Months	4 Months	3 Months	5 Months	11 Months	11 Months	11 Months	3 Months	4 Months	4 Months	2 Months	3 Months	4 Months
Type of room	Double room	Quad room	Triple room	Double room	Triple room	Triple room	Quad room	Quad room	Triple room	Triple room	Double room	Quad room	Quad room	Triple room	Triple room	Double room

The views and experiences of participants during relocation to the LTC facility reflected four interrelated key themes: wish to minimize the burden, but stay connected with the family; perceived barriers to adaptation; valuing tailored care; and acceptance and engagement. Each of these themes included several interrelated subthemes that influenced one another and represented the different stages in their personal relocation journey although there was no clear sequential order between the themes. These themes are presented in detail below supplemented with extracts from interviews.

### Wish to minimize the burden, but stay connected with the family

The participants described how they experienced conflicting decisions between their desire to continue living in their family homes which they perceived as ideal and the need to avoid disrupting the lives of their families whom they depended upon for the activities of their daily living. Most participants viewed the relocation to the care facility as a way of minimizing the burden of their care from family members so that family members can continue with their own commitments without worrying about caring for them:

“I used to be in day care, but when I went back home at night, no one was home. My son and daughter-in-law are both very busy. Sometimes they go to Shanghai. Therefore, I can only live here.” (R11, Male, 87 years, living in the facility for 3 months)

However, they still desired to keep the connection with their family members and friends. In order to avoid alienating themselves from those who they were emotionally close, participants usually chose facilities in places nearby to where their family members or friends were residing or working:

“My daughter works nearby. She chose this place so she could visit me easily, even during her break at noon.” (R6, Female, 80 years, living in the facility for 3 months)

“I came here because I had a distant relative who was the head nurse here. She said it wasn’t bad here and said I should tell my grandson to bring me here.” (R2, Male, 88 years, living in the nursing home for 8 months)

There was a strong desire among almost all participants not to feel abandoned by their family members. There was also constant reflection of how much they cherished visits from family members and how important it was for them to continue receiving attention from family members who cared for them in the past:

“My family will come every day, they come here after they work at night, they will accompany me and chat with me, bring some food for me” (R2, Male, 88 years, living in the facility for 8 months)

“Whenever my daughter come, she will bring me to the garden at the back to walk, being in the sun, this is very good!” (R6, Female, 80 years, living in the facility for 3 months)

Some participants wished that their partner or spouse came and lived in the facility to keep the intimacy and connection:

“I am thinking that, if my wife comes and lives with me after she retires a few years from now, we can live here together as couple. I really wish my wife can live with me in the future so I can have company.” (R4, Male, 69 years, living in the facility for 10 months)

A few participants made active efforts to interact with other residents in the LTC facility with the view of making friendships in their new environment. Often there was a feeling that they have shared experiences and they would be able to understand each other’s concerns:

“I have made friends here! That old friend is really nice; everyone is very happy. Those elderly that been through pain, will then cherish the blessing, and can be nice with others.” (R8, Female, 86 years, living in the nursing home for 11 months)

### Perceived barriers to adaptation

While there were varying reasons for relocation to the LTC facility including lack of family support and inability to perform self-care, the participants often experienced psychological resistance while making the decision to leave home and move to the facility. Fear of losing their autonomy and the ability to perform self-care was a major reason for the resistance to adapt to care facilities.

“After my stroke, I also thought about moving around to restore my mobility. But after I entered the facility, the environment was limited, and I was unable to practice walking.” (R1, Male, 83 years, living in the nursing home for 5 months)

Dislike or lack of trust in the care provider and facility inadequately meeting the care needs was also a perceived barrier running through the relocation process.

“When I came to this facility, my experience with the care providers was similar to my previous experience. I cannot trust those who take care of me.” (R5, Female, 79 years, living in the facility for 4 months)

Individual emotional reactions to new environments acted as perceived barriers to adaptation to the LTC facility environment for many participants. These emotional reactions were manifested in different ways. Many of the participants, especially those who have had physical and mental health issues, feared of being neglected and were concerned about their overall safety from injuries and accidents especially when they first arrived at the LTC facility:

“When I first came here, I didn’t know anyone, and I was afraid my family would neglect me. I am afraid the people here won’t take care of me, and I am afraid of falls.” (R3, Female, 88 years, living in the facility for 9 months)

Participants who struggled to adapt also expressed feelings of missing their home and their families, and their persistent desire to return home:

“When I first got here, I definitely missed home! When I miss home, I cry! I feel like I am just waiting to die!” (R9, Female, 90 years, living in the facility for 11 months)

The unfamiliar or uncomfortable environment of the LTC facility proved to be a shock for some participants in the initial months of their stay in the facility. Their perceptions of the new environment had a large impact on some participants who struggled to adapt to the differences between the facility and home. While all participants lived in shared rooms, they often felt they were living with strangers and reported problems such as difficulty sleeping due to disturbances from their roommates:

“Of course, when I first got here, I had difficulty adjusting to living here. I have many family members in my home, so it is very lively. When I got here, each person only has a bed. I didn’t know the people beside me, and I didn’t talk to them. I was very depressed and felt alone. During the next 3 days, I cried whenever I thought about my situation. (R6, Female, 80 years, living in the facility for 3 months)

“Some people here moan and make noise. Sometimes it is so noisy, I can’t sleep at night. The caregivers don’t have good manners. They chat loudly, even at night.” (R1, Male, 83 years, living in the nursing home for 5 months)

### Valuing tailored care

The participants considered the provision of tailored care - including helping them to take their medication regularly, taking them to the doctor if needed, and preparing special meals according to their preferences - as valuable in their adaptation to the facility:

“I am a devoted Buddhist. They prepare vegetarian food for me every morning.” (R11, Male, 87 years, living in the facility for 3 months)

“I like to drink coffee. I need to drink many cups of coffee a day. They even help me buy more coffee when my coffee is finished.” (R4, Male, 69 years, living in the facility for 10 months)

Most of the participants valued not only the tailored physical care but also the emotional care that they received at the LTC facility. They recounted how they appreciated having nursing staff available to provide care and comfort suited to their individual needs:

“If you have a stomach ache, the nursing staff will come and show concern, console you, and take care of you.” (R8, Female, 86 years, living in the nursing home for 11 months)

Participants who were able to perform self-care appreciated the recognition from the nursing staff of their ability to perform self-care activities:

“I had my artificial anus for more than ten years. I’ve used different types, such as a clip and a stick. I don’t need them to teach me. All I need is scissors, and I can cut ostomy bag myself. The nurses said I am very good. They all said they will use my way to change ostomy bag. They say I am a professional! (laughs)” (R1, Male, 83 years, living in the nursing home for 5 months)

Most of the participants expressed appreciation for the nursing staff who showed concern and intimacy:

“All of their services are really good. They don’t get angry at us. We are all very harmonious. The nurses are really nice. They are nice to me. They greet me, and if I have any needs, they come and help me handle it quickly.” (R10, Female, 77 years, living in the facility for 11 months)

“They are like my grandson: very cordial and polite” (R9, Female, 90 years, living in the facility for 11 months)

### Acceptance and engagement

Participants who lived relatively longer in the facility appeared to be more positive in their views about their life in the LTC facility. There were recurring views among these participants that they were living in a safe environment. They perceived that the physical infrastructure of the LTC - including vacant space to walk around and having railing to hold on to - was tailored to the needs of the elderly and this was seen to be important to keep them safe from falls and injuries:

“Environment is very safe, room, bathroom, any place is all flat, there are fence on the side that you can support on. I haven’t [had a] fall here, safety is what I think done very good in this place.” (R2, Male, 88 years, living in the nursing home for 8 months)

The availability of staff to call for in the event of an injury or a fall was another factor that was perceived as crucial. The provision of various activities that are interesting to elderly and the support to participate in these activities was seen as a major factor behind the acceptance of the facility:

“There are more people here, they hold activities every day, have karaoke, have big television, have Bingo, have sport, many types of activities, make the elderly feel interested.” (R4, Male, 69 years, living in the facility for 10 months)

The comments of some participants reflected their decision to face the challenge of relocation by trying to accept their stay in the facility and to adapt themselves to the new environment due to their needs for constant care. As a mark of successful adaptation and assimilation, they remarked that they saw the LTC facility as their second home and envisaged themselves to be living in the facility for the rest of their lives:

“I am very used to living here. It’s just like my home. I even thought of staying here during new year! It’s more lively here!” (R8, Female, 86 years, living in the nursing home for 11 months)

“When I came, I found they can provide me meal and insulin injection regularly, and the risk of coma (due to hypoglycemia) could be prevented. So I found stay here wasn’t bad, and better safety than home. I thought I should try it and see if I like it. So, I haven’t left!” (R7, Female, 89 years, living in the nursing home for 5 months)

“I had a stroke, which was very inconvenient for me. I am afraid that I cannot do activity of daily life by myself. After I got discharged from the hospital, my family took me here. In this setting, they provide care for me and prevent accidents happen to me. I need to stay here, and it isn’t bad for me and my family!” (R9, Female, 90 years, living in the facility for 11 months)

## Discussion

This paper builds on the evidence on the relocation experiences of the elderly to LTC facilities and explores in-depth the day-to-day experiences in the first 12 months following the relocation to the facility in Taiwan. As qualitative studies in Taiwan are relatively sparse on the topic, we have used existing studies from the country as well as the international literature to compare and contrast the findings as discussed in the following sections.

Four interrelated themes emerged in the study with respect to the experiences of adapting and settling in to the LTC facility: wish to minimize the burden, but stay connected with the family; perceived barriers to adaptation; valuing tailored care; and acceptance and engagement. Researchers have argued that relocation is a major life change for most elderly people [40] and the accounts from participants in our study supported this view. In many cases, elderly people do not choose to live in LTC facilities voluntarily and as such may not be prepared for the change in circumstances [32, 41, 42].

For the participants in our study, the decision to move to the LTC facility was mainly driven by the desire to avoid disrupting the lives of their family members, whom they were dependent upon, and this appeared to be a key motivating factor for their adaptation the life in LTC facility. All participants had long-term illnesses or health conditions that required access to round-the-clock care including provision of necessary medications and support for preventing falls and injuries, which were beyond the capacity of their family members to provide. Previous studies among Chinese elderly living in LTC facilities have shown that they were willing to compromise their own desires and wishes to maintain family harmony and they continued living in the facility hiding their own feelings and emotions even if they were unhappy [41]. However, the notion of ‘forced choice’ as reported by family members of the elderly in Taiwan was less explicit in the accounts of our participants [34].

Relocation to the LTC facility often resulted in the elderly to feel “cut off the root” as they left their homes, family members and friends [35]. The participants in our study, desired to keep the connection with their friends and family members and to avoid alienating themselves from those who they were emotionally close. They choose LTC facilities nearer to their family home to keep this connection and to increase the frequency of family members’ visits. Continued relationships with family members and significant others is a key factor reported in studies internationally in facilitating transition to LTC facilities [12, 34, 42–44]. Intimate interaction and communication with close relatives or friends diminished the feelings of isolation and loneliness experienced by the elderly when they moved into the facility [35, 45]. This is of particular significance in the family-oriented culture of the Taiwanese society. The wish for their partner or spouse to come live in the facility and their efforts to communicate and interact with other residents in the LTC facility with the view of making friendships also strongly indicate the wish to avoid alienation.

Consistent with findings from other studies, we found that the barriers experienced by the elderly when they relocated to LTC facilities could originate from different factors [17, 45, 46]. The elderly in our study felt threatened by their loss of autonomy and their ability to perform self-care; feeling of dislike and distrust of the care provider and facility; fear of neglect and concerns about overall safety from injuries and accidents; feelings of missing their home and families; unfamiliar or uncomfortable environment. The need for approaches to care in LTC facilities that facilitate autonomy whilst encouraging self-care has been highlighted in studies internationally [11, 47]. The feelings of dislike and distrust of the care provider and facility and concerns about being neglected or being unsafe in the facility could be linked to negative perceptions of facility based care more generally. As LTC facilities offer a relatively new care model in Taiwan, there may be very little awareness among the elderly population in general about facility based care, and the elderly may find it difficult to develop a liking for the facility environment that may be perceived as strange or to have trust in the care provided by strangers. Although less explicit in the accounts of our participants, uncaring conversations by staff [48] and a feeling of being forced to be co-operative with staff [8] have been indicated as hindering factors for successful transition, and it has been suggested that sensible symbols will help foster the elderly’s trust in the care provided [49]. Researchers have indicated that the physical design of the LTC facility influenced the residents’ activities and interactions [50], and the design could be a hindrance or facilitator for smooth transition by way of establishing connections and relationships [51]. The design of the care facility could also enable the elderly to pursue their interests and participate in activities and to have a sense of calm and peace [38, 44, 50]. All our participants lived in shared rooms, but they wished for more personal space and less disturbance from their co-residents.

While the unfamiliar or uncomfortable environment of the LTC facility was a perceived barrier for adaptation for participants in the initial months, those who lived longer in the facility appeared to consider the LTC as a safe environment offering protection from falls and injuries along with the availability of staff to call upon in the event of an injury. While the views from our participants confirmed the important role that the elements of the design and physical infrastructure of the LTC facility played in the successful adaptation of the residents at various time points, the differing views in this regard indicated the need for further detailed longitudinal investigation in this area.

Overall, the perceived barriers to adaption reported by our participants suggest that allowing elderly people to choose the LTC facility, and, wherever possible, respecting their wishes to maintain autonomy in their day to day care and fostering trust in the care providers will have positive impacts on their adaptation to living in the facility. Although we were unable to draw clear patterns due to the relatively small samples, we noted that participants who lived in the facility longer expressed more positive responses.

While the fear of losing autonomy was a perceived barrier for successful adaptation as mentioned before, the provision of tailored care in accordance with individual needs was an aspect that was highlighted in our study as valuable to facilitate adaptation to the facility. This is in concurrence with conclusions of studies from Taiwan and other countries that approaches to care that did not consider individual preferences and the uniqueness of residents could be potential barriers to successful transition [12, 34, 52]. LTC facilities should provide tailored care to assist residents in integrating [35, 53]. Care providers assisting the elderly in their transition to a LTC facility tended to focus more on technical aspects, such as the physical aspects of the environment or the availability of equipment, rather than on psychological aspects such as the shock and distress that can be induced by major changes in living conditions following relocation [54]. Our findings showed that in order to support older adults to adapt and integrate into an unfamiliar facility environment, the nursing staff as well as other care providers must provide appropriate emotional and supportive care. Lee (2010) reported the link between greater perceived emotional support from staff and better adjustment to relocation [26]. Other studies have pointed out the role of interpersonal relationships between the elderly residents and the LTC staff as a potential barrier or facilitator for successful relocation to the LTC facility [12, 27, 55]. Alongside the provision of professional health care, aspects such as cordial greetings, proactive enquiries about physical comfort were all perceived as valuable in alleviating the fears and anxieties of the elderly and to help them feel relaxed and safe in LTC facilities [53, 54, 56]. Researchers have suggested that evidence based staff training programmes aimed at advancing further understanding and enhancement of the relocation transition that enables choice, independence and self-identity for the elderly should be a component of intervention programme [12].

The interest of our participants in leisure time activities indicated the importance of integrating and supporting active participation in individual and group based activities in an appropriate manner as part of their daily routine. Previous studies have argued that elderly people often expected caregivers to provide a convenient friendly environment with activities that were designed to support them physically, emotionally and spiritually ensuring that they were supported to lead a life that is meaningful [45]. Relocation to the LTC facility can be considered successful when the resident considers it as a home [57]. As a mark of successful adaptation and assimilation, some participants in our study viewed the LTC facility as their second home and envisaged themselves to be living in the facility for the rest of their lives. The perceptions of the LTC facility as ‘a new home’ or ‘almost like home, but without their families’ has been reported in previous studies implying that ‘home’ was viewed as a quality within the facility implying a home-like place to live [9, 12, 58].

The study is one of the very few recent qualitative studies conducted on this topic in Taiwan and offer relevant insights to a range of stakeholders including practitioners, policy makers and future researchers. The qualitative methodology underpinned by a grounded theory approach facilitated the participants to freely express their experiences and views and enabled the researchers to deconstruct the meanings that are associated with the narratives. The study’s scientific rigor was enhanced through a number of measures as described in the methodology section. The study has certain limitations, however. As a qualitative study with a relatively small sample of 16 participants, the findings may not represent the views of all the elderly who have relocated to LTC facilities in Taiwan or elsewhere. The study only explored the experiences of those who had lived in the LTC facility for a period of 12 months or less with the majority of participants living in the facility for less than 6 months. Inadvertently, the sample comprised of more female participants with the majority aged over 80 years although there was no explicit indication of participants’ age affecting their perspectives.

## Conclusion

With a growing ageing population, relocation of the elderly to LTC facilities appear to evolve as a key future trend in the care of the elderly. Supporting the elderly and their families towards making a peaceful and smooth transition is of considerable importance to health and well-being and our findings provide useful insights for policy and practice in this regard. Our findings suggest that relocation to the LTC facility is a dynamic process in the first year of their life in the facility, and involve a range of emotions, feelings and experiences starting from the time the decision is made. The adaptation of the elderly to the LTC facility can be maximized if the relocation is well planned with sustained opportunities to maintain involvement of the family members in care provision.

The relocation program should include a carefully formulated tailored care plan taking into account individual care needs based on discussions with the elderly people themselves and their family members. The plan should include identification of care needs based on detailed physiological and psychological assessments and considerations of unique personality characteristics and individual needs. This should be followed by continuous evaluation of changing needs once the elderly move into the facility. They should also be supported and encouraged to partake in day-to-day activities of the LTC facility including rehabilitative, restorative, and entertainment activities. It is likely that many elderly need round-the-clock care and this needs to be taken into active consideration while choosing the facility and in care planning. Moreover, adaptation of the elderly into the LTC facility can be maximized if the relocation is well planned with provisions for individually tailored care and family involvement.

Overall, although relocation to an LTC facility is challenging for most older adults, the elderly whose physical and psychological needs can adequately be met through relocation can benefit from this challenge if they integrate well and view the facility as another home. Further long term studies are need to explore the differing needs and experiences of the elderly at different stages in their lives as well as the nature of interventions to support ongoing interactions and engagement with the family members. Development and evaluations of interventions to support LTC staff to have positive relationships and meaningful interactions with the elderly as well as longitudinal investigations of how the design of the LTC facility facilitates or hinders adaptation are other areas for future research.

## Supplementary information

**Additional file 1.** Interview guide.

## Data Availability

The datasets used and/or analysed during the current study are available from the corresponding author on reasonable request.

## Abbreviation

LTC: Long-Term Care
