# Supplementary material for: Relocation experiences of the elderly to a long-term care facility in Taiwan: a qualitative study
Source: BMC Geriatr. 2020 Aug 6;20:280. doi: 10.1186/s12877-020-01679-5 (PMC7409483; doi:10.1186/s12877-020-01679-5)
Supplement: Supplementary file 1 — Additional file 1. Interview guide. [file 12877_2020_1679_MOESM1_ESM.doc]

**Interview guide**

*Greeting and introduction. Explanation of the interview process and recording consent*

1. Please tell me what are reasons for you to move to the long-term care facility/nursing home?
2. Please tell me about your experiences of relocating to the facility
3. Please tell me your daily life /activities in a long-term care facility/nursing home?
4. Please tell me what and why you enjoy to live in a long-term care facility/nursing home? And what you do not like to do or not enjoy to live in a long-term care facility/nursing home?
5. What are the reasons for you continued to live in this long-term care facility/nursing home?
